# Nutrient acquisition efficient rootstocks improve zinc nutrition of top-grafted citrus trees on calcareous soil

**DOI:** 10.3389/fpls.2025.1615405

**Published:** 2025-07-31

**Authors:** Jiawei Xie, Huaye Xiong, Runzheng Niu, Yuheng Wang, Yuehong Wang, Mohammad Naeem Lali, Jingkun Zhao, Xiaojun Shi, Heinz Rennenberg

**Affiliations:** ^1^ Center of Molecular Ecophysiology (CMEP), College of Resources and Environment, Southwest University, Chongqing, China; ^2^ Interdisciplinary Research Center for Agriculture Green Development in Yangtze River Basin, College of Resources and Environment, Southwest University, Chongqing, China; ^3^ Hechuan District Grain and Oil Development Guidance Station, Chongqing, China; ^4^ Department of Forestry and Natural Resources, Faculty of Agriculture, Bamyan University, Bamyan, Afghanistan; ^5^ Chongqing Agro-Tech Extension Station, Chongqing, China; ^6^ Chair of Tree Physiology, Institute of Forest Sciences, Albert-Ludwigs-Universität Freiburg Georges-Köhler-Allee 53/54, Freiburg, Germany

**Keywords:** citrus, top grafting, rootstock, zinc deficiency, photosynthetic efficiency, carbon and nitrogen partitioning, metabolome composition

## Abstract

**Introduction:**

Zinc deficiency is a common issue in top-grafted citrus trees. Citrus scions top-grafted on rootstocks may exhibit zinc deficiency due to reduced mineral absorption and transport. Therefore, selecting the appropriate rootstock is thought to be crucial for Zn nutrition of top-grafted citrus trees.

**Methods:**

To test this assumption, we performed top-grafting of citrus scions using *Poncirus trifoliata* (L.) Raf. and *Citrus junos* (Sieb.) Tanaka as rootstocks and analyzed growth, Zn nutrition, and physiological traits of the top-grafted citrus trees.

**Results and discussion:**

The results indicated that, compared with the *Poncirus trifoliata* (L.) Raf. rootstock, the *C. junos* (Sieb.) Tanaka rootstock significantly increased the Zn level in new leaves, mature leaves, stems, and roots by 81.69%, 66.18%, 97.52%, and 45.94%, respectively, and positively influenced growth, photosynthetic efficiency, and foliar carbon and nitrogen metabolite concentrations in the top-grafted citrus trees. Metabolome analysis of leaves revealed that intermediates of the glyoxylate, dicarboxylate, ascorbate and aldarate metabolic pathways were responsive to different Zn levels. Thus, *C. junos* (Sieb.) Tanaka rootstock stimulated plant growth, boosted Zn acquisition, and enhanced the physiological performance of top-grafted citrus plants. The mechanisms by which *C. junos* (Sieb.) Tanaka rootstocks improve the performance of citrus plants require further research.

## 1 Introduction

Zn, serving as an indispensable trace element for plant growth and development, fulfills various functions within plants, i.e., enzyme activation (Saleem et al., 2022), facilitation of protein synthesis (Kasiviswanathan et al., 2024), regulation of hormone metabolism (Chattha et al., 2022), promotion of photosynthesis (Jocsak et al., 2024), and enhancing stress resistance (Kuvelja et al., 2024; Sofy et al., 2020). In recent years, Zn deficiency in plants has become an increasingly recognized issue in agriculture, casuing plant growth and yield reduction (Lilay et al., 2021), which in turn affects human food supply and health (Khan et al., 2022). Zinc deficiency has been reported in various food crops, such as wheat, rice, maize and soybeans (Shukla et al., 2022; Yogi et al., 2023; Xu et al., 2021; Martínez Cuesta et al., 2023). However, studies focusing on Zn deficiency in fruit trees are still limited. This gap in research highlights the need for investigations of the effects of Zn deficiency on fruit tree growth and productivity.

Citrus is one of the most important industrial crops in the world and occupies an important position in the global fruit market (Rao et al., 2021). However, citrus commonly suffers from Zn deficiency on calcareous soils (Younas et al., 2023), which poses a widespread challenge to citrus cultivation, impacting tree health, damaging cytology and morpho-anatomy, reducing flowering and fruit set, diminishing fruit yield, and compromising fruit quality, ultimately leading to economic losses in horticulture (Xing et al., 2016). At the high pH of calcareous soils, Zn is bound to carbonates, forming insoluble compounds (Natasha et al., 2022), which decreases the solubility and plant availability of Zn in the soil. Zn deficiency symptoms may be induced under these conditions. Under such conditions, citrus trees may exhibit Zn deficiency symptoms, including interveinal chlorosis as well as smaller, narrower, and thinner leaves (Fu et al., 2016). To address this problem, it has been suggested to alleviation Zn efficiency in citrus by applying soil fertilizers, foliar Zn sprays, and utilizing chelated Zn (Boaretto et al., 2024; El-Gioushy et al., 2021). However, even on Zn-deficient soils, the extent of Zn deficiency can differ among citrus trees of the same variety (Toplu et al., 2010). This difference is reflected by varying Zn absorption, utilization, and distribution efficiencies between different plants. Heterogenity in rhizosphere microbial communities (Chen et al., 2017), soil moisture (Tadayon, 2020), and mineral soil nutrient composition may be responsible for differences in the availability of Zn in the soil to plants (Srivastava and Singh, 2005), leading to variations of Zn nutrition among citrus plants of the same variety in Zn-deficient soils. In addition, graft compatibility directly influences the efficiency of nutrient exchange between scions and the rootstock (Nawaz et al., 2016). Poor graft compatibility can reduce the efficiency of Zn transport across the graft interface, causing the scion to fail in acquiring sufficient amounts of Zn (Khankandani et al., 2019), thereby leading to severe Zn deficiency symptoms. Furthermore, the selection of rootstock has a significant impact on the scion’s Zn absorption capacity (Ikinci et al., 2016). Different citrus rootstocks exhibit varying efficiencies in Zn uptake and transport, potentially regulating Zn availability by affecting root morphology, growth, and the microenvironment of the rhizosphere (Ghimire et al., 2023; Chen et al., 2014; Toplu et al., 2012).

Top grafting as a common agricultural practice combines three distinct citrus species (Rootstock-interstock-scion) to create a new hybrid with favorable traits such as crown formation, rapid seeding, and high fruit quality and yield (Calderón et al., 2021; Wang et al., 2020). For Zn deficiency in top-grafted citrus trees, graft incompatibility does not seem to be the main factor, as the same citrus variety grafted on the same interstock shows different degrees of Zn deficiency. In fact, interstocks play a crucial role in modulating plant growth, enhancing fruit quality, and mitigating incompatibility between rootstock and scion (Rong et al., 2023). It can alter the stem structure of grafted plants and serves as a critical determinant of nutrient and water transport efficiency (Yu et al., 2025). However, studies on top-grafted fruit trees have shown that the use of an interstock may increase the transport distance of mineral nutrients from the root to the scion, resulting in nutrient loss and elevated metabolic demands during transport (Zhou et al., 2023). In this context, nutrient acquisition efficient rootstocks becomes a key factor influencing Zn deficiency in citrus. Therefore, Zn-efficient rootstocks can be used to address Zn deficiency in top-grafted citrus on calcareous soils.

Among the citrus rootstock varieties, *Poncirus trifoliata* (L.) Raf. and *Citrus junos* (Sieb.) Tanaka are widely used in Chinese citrus orchards (Wu et al., 2019). Still, in alkaline soils, when *Poncirus trifoliata* is used as a rootstock for citrus, the absorption rates of nutrients such as Zn and iron are very low, resulting in nutrient deficiencies and stunted growth. However, *C junos* can absorb these elements at relatively higher levels, thereby maintaining healthy growth (Chun et al., 2020). Currently, most studies focus on simple rootstock-scion combinations, whereas investigations into how rootstocks influence Zn nutrition in top-grafted citrus trees under calcareous soil conditions remain limited. In addition, although differences in Zn uptake capacity among rootstocks have been previously reported, the mechanisms by which rootstocks regulate Zn transport and influence carbon and nitrogen metabolism in top-grafted citrus trees remain unclear. In this study, we integrated nutrient, physiology, and metabolomics to systematically assess these effects. We hypothesize that (i) differences in rootstocks can significantly affect growth and Zn nutrition of top grafted citrus plants; (ii) the photosynthesis of citrus leaves is significantly affected by the application of different rootstocks, thereby changing carbon and nitrogen partitioning; (iii) differences between rootstocks lead to difference characteristic metabolite compositions in metabolic pathways of citrus leaves by the interaction with Zn deficiency. With this information we intent to provide a theoretical basis for improving the current production of citrus on calcareous soil.

## 2 Materials and methods

### 2.1 Plant materials

The present experiment was conducted at the experimental station of Southwest University (30°26′ N, 106°26′ E) in Beibei, Chongqing, China, from March 2022 to May 2023. Two-year-old citrus trees of *Citrus sinensis* (L.) Osbeck grafted on *Poncirus trifoliata* (L.) Raf. and *Citrus junos* (Sieb.) Tanaka rootstocks were planted in February 2022 in plastic pots with a top and bottom size of 38 and 30 cm diameter, respectively, and 40 cm height. The scion was *Citrus reticulata* Ehime No. 38. For potted plant growth, an alkaline purple soil with pH 7.25, organic matter concentration of 9.85 g kg^-1^, plant available nitrogen of 44.2 mg kg^-1^, plant available phosphorus of 11.2 mg kg^-1^, plant available potassium of 172 mg kg^-1^, exchangeable calcium of 2921 mg kg^-1^, exchangeable magnesium of 125 mg kg^-1^, plant available copper of 0.83 mg kg^-1^, plant available iron of 17.3 mg kg^-1^, plant available manganese of 24.6 mg kg^-1^, plant available boron of 0.87 mg kg^-1^ and plant available Zn of 0.42 mg kg^-1^ was used.

### 2.2 Experimental treatments

**Figure 1:**
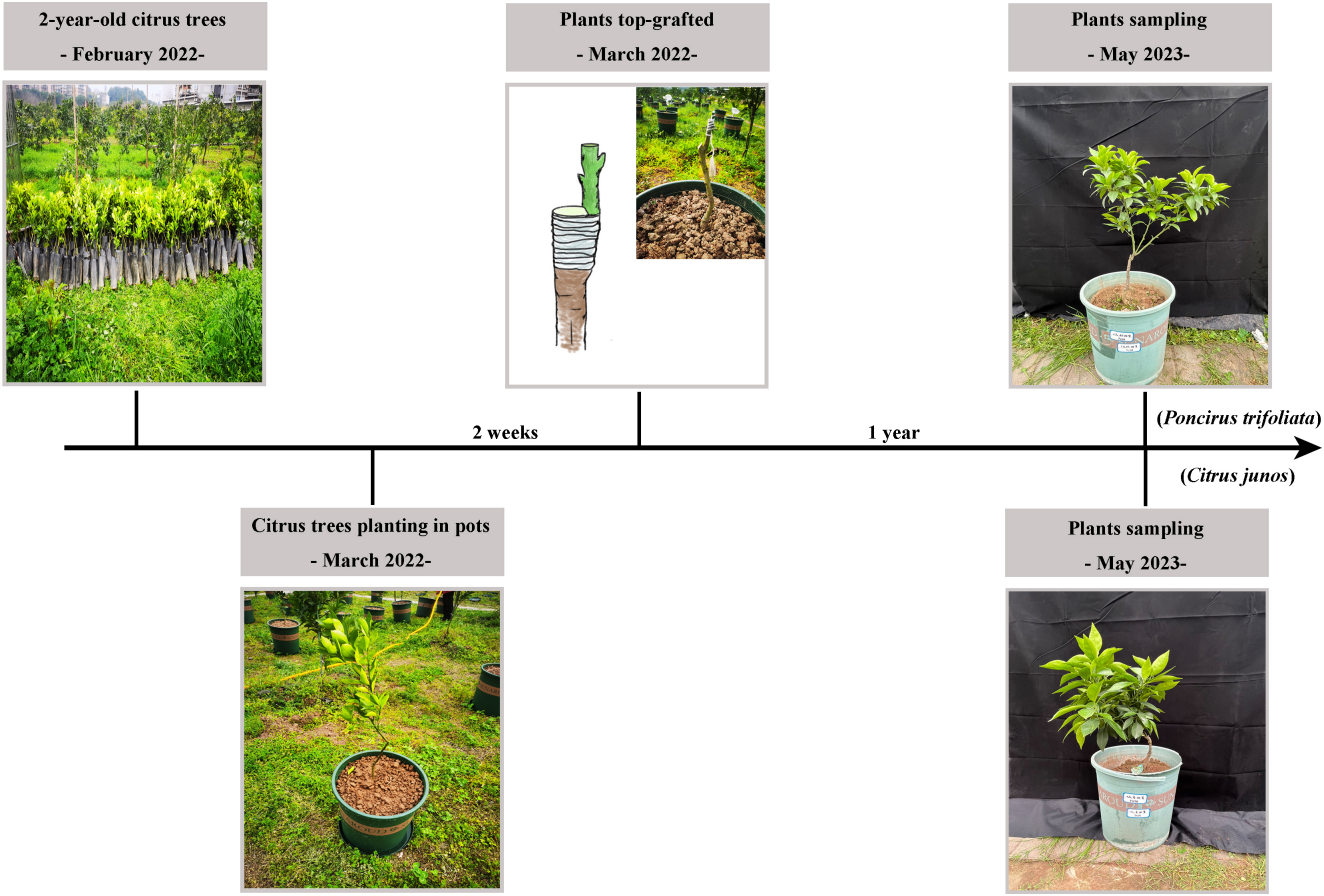
Overview of the experimental design. In March 2022, two-year-old citrus trees with two different rootstocks were planted in plastic pots. Then, all citrus trees were top grafted with scions of *Citrus reticulata* Ehime No. 38. In May 2023, top-grafted plants were sampled.

All citrus trees were top grafted in March 2022 with scions of *Citrus reticulata* Ehime No. 38 and sampled in May 2023 (**Figure 1**). Fertilizer applications were 200 mg N kg^-1^, 90 mg P_2_O_5_ kg^-1^, 150 mg K_2_O kg^-1^, and 25 mg Zn kg^-1^ in 2022 and 50 mg N kg^-1^, 90 mg P_2_O_5_ kg^-1^, 150 mg K_2_O kg^-1^, and 10 mg Zn kg^-1^ in 2023. The fertilizers used for these amendments were urea, superphosphate, potassium sulfate, and EDTA-Zn.

### 2.3 Plant biomass measurements

The sample collection for biomass measurements was conducted in May 2023, when the leaves of the spring shoots were fully mature. For this purpose, citrus trees were harvested, cleaned from soil, divided into young leaves, mature leaves, stems, and roots. All samples were dried at 105°C for 30 min, and then at 65°C to constant weight for dry weight determination. The shoot biomass was calculated as the sum of the dry masses of young leaves, mature leaves and stems. The total biomass was calculated as the sum of shoot and root biomass. Samples collected from individual plants were treated as one biological replicate, and three biological replicates were collected for each treatment. For physiological and metabolomic analyses, 5 young leaves and 5 mature leaves were collected from each plant.

### 2.4 Determination of Zn concentration

The Zn concentrations of young leaves, mature leaves, stems and roots were determined by ICP-OES (ICP-OES5110010499, Agilent Technologies, USA) analysis after microwave digestion of tissue samples with HNO_3_-H_2_O_2_ (Wang et al., 2023).

### 2.5 Measurement of gas exchange parameters

At clear weather conditions, the Li-6400 portable photosynthesis measurement system (Li-Cor, Inc., Lincoln, Nebraska, USA) was used for the determination of H_2_O and CO_2_ gas exchange of the leaves. The parameters inside the leaf cuvette were set as follows: photosynthetically active radiation (PAR) at 1200 μmol m² s^-1^, CO_2_ concentration at 400 μmol mol^-1^, temperature at 30°C, and relative humidity between 45% and 65%. Measurements included net photosynthetic rate (P_n_), intercellular CO_2_ concentration (C*_i_*), stomatal conductance (*g*_s_), and transpiration rate (T_r_).

### 2.6 Physiological analyses

Young leaves (from spring branches) and mature leaves (from last year’s autumn branches) were collected, snap-frozen in liquid N_2_ and stored at -80°C until further analyses. Before analyses, leaf samples were ground by mortar and pistil under liquid N_2_. Chlorophyll a, chlorophyll b, and carotenoids were extracted from 0.1g fresh leaves with 80% acetone, and the absorbance of the extracts was measured at 665, 645, and 470nm with a spectrophotometer (Xiong et al., 2022). Soluble sugar, starch, soluble protein and free amino acid concentrations were determined with commercial test kits (Nanjing Boyan Biotechnology Co., Ltd., Nanjing City, Jiangsu Province, China), according to the manufacturer’s instructions. The anthrone method was applied to measure soluble sugar and starch at 620 nm absorbance (Feng et al., 2023), using 0.1g fresh leaves. For the determination of soluble protein and free amino acid concentrations (Song et al., 2015; Hameed et al., 2023), 0.1g leaf samples were ground at 4 °C and the supernatant was collected after centrifugation at 8000 g and 4 °C for 15 min. The absorbance of the extract was determined at 562nm and 570nm for soluble protein and free amino acid quantification, respectively. All standard substances used for detection were provided by the purchased assay kits.

### 2.7 Untargeted metabolome analyses

#### 2.7.1 Sample preparation

Aliquots of 50 mg leaf tissue powder were added to 1000 μL of extraction solution (methanol, acetonitrile, water, 2:2:1, 2 mg/L internal standard) and vortex for 30 seconds. Ceramic beads were added, the samples grinded at 45 Hz for 10 min, sonicate in an ice-water bath for 10 min, and incubated at -20°C for 1 h. Subsequently, samples were centrifuged at 4°C and 14009 xg for 15 min, 500 μL of supernatant transferred to an Eppendorf (EP) tube, and dried in a vacuum concentrator (CV200, Beijing JM Technology Co., Ltd., Beijing, China). Samples were dissolved with 160 μL extraction solution (acetonitrile, water, 1:1), vortexed, sonicated for 10 min, centrifuged again, and 120 μL supernatant were transferred to a UPLC vial. An aliquot 10 μL of each sample was used for quality control analysis.

#### 2.7.2 UPLC-MS/MS analysis

The Liquid Chromatography-Mass Spectrometry (LC-MS) system used for metabolome analyses consists of a Waters UPLC Acquity I-Class PLUS coupled with a Waters Xevo G2-XS QTOF high-resolution mass spectrometer (Waters Corporation, Milford, USA). The column used for metabolite separation was a Waters Acquity UPLC HSS T3 column (1.8 μm, 2.1*100 mm). In positive ion mode, mobile phase A consisted of 0.1% formic acid in water, mobile phase B of 0.1% formic acid in acetonitrile. In negative ion mode, mobile phase A consisted of 0.1% formic acid in water, and mobile phase B of 0.1% formic acid in acetonitrile. The liquid chromatography gradient parameters were as follows: 0-10 min, 98-2% A; 13-15 min, 2-98% A, with an injection volume of 1 μL. The electrospray ionization (ESI) ion source parameters were: capillary voltage: 2000V (positive ion mode) or -1500V (negative ion mode); cone voltage: 30V; ion source temperature: 150°C; gas temperature: 500°C; cone gas flow: 50 L/h; gas flow: 800 L/h.

#### 2.7.3 Metabolome data analyses

After data conversion with metaX software, principal component analysis (PCA) and partial least squares discriminant analysis (PLS-DA) were conducted to obtain the VIP values for each metabolite. Statistical significance (*P* value) and fold change (FC) in various groups were determined using the t-test (Zhao et al., 2023). Metabolites with VIP > 1, *P* value < 0.05, and FC ≥ 2 or FC ≤ 0.5 were identified as differentially expressed. Correlation analysis (Pearson correlation coefficient) was performed using R, with the significance level set at *P* < 0.05. Differential metabolites were annotated via the KEGG database (https://www.genome.jp/kegg/pathway.html).

### 2.8 Statistical analysis

The Shapiro-Wilk test indicated that data for all plant parameters were normally distributed (*P* > 0.05). Levene’s test confirmed homogeneity of variances between the groups (*P* > 0.05). Therefore, independent sample *t*–test was used to analyse the effect of the two rootstocks on plant parameters (*P* < 0.05). All statistical analyses were performed using SPSS version 16.0 (IBM Corp., Armonk, NY, USA). Graphical analysis was performed using the origin 2018 software (OriginLab Corp., Northampton, MA, USA).

## 3 Results

### 3.1 Effects of different rootstocks on the growth of top grafted citrus

**Figure 2:**
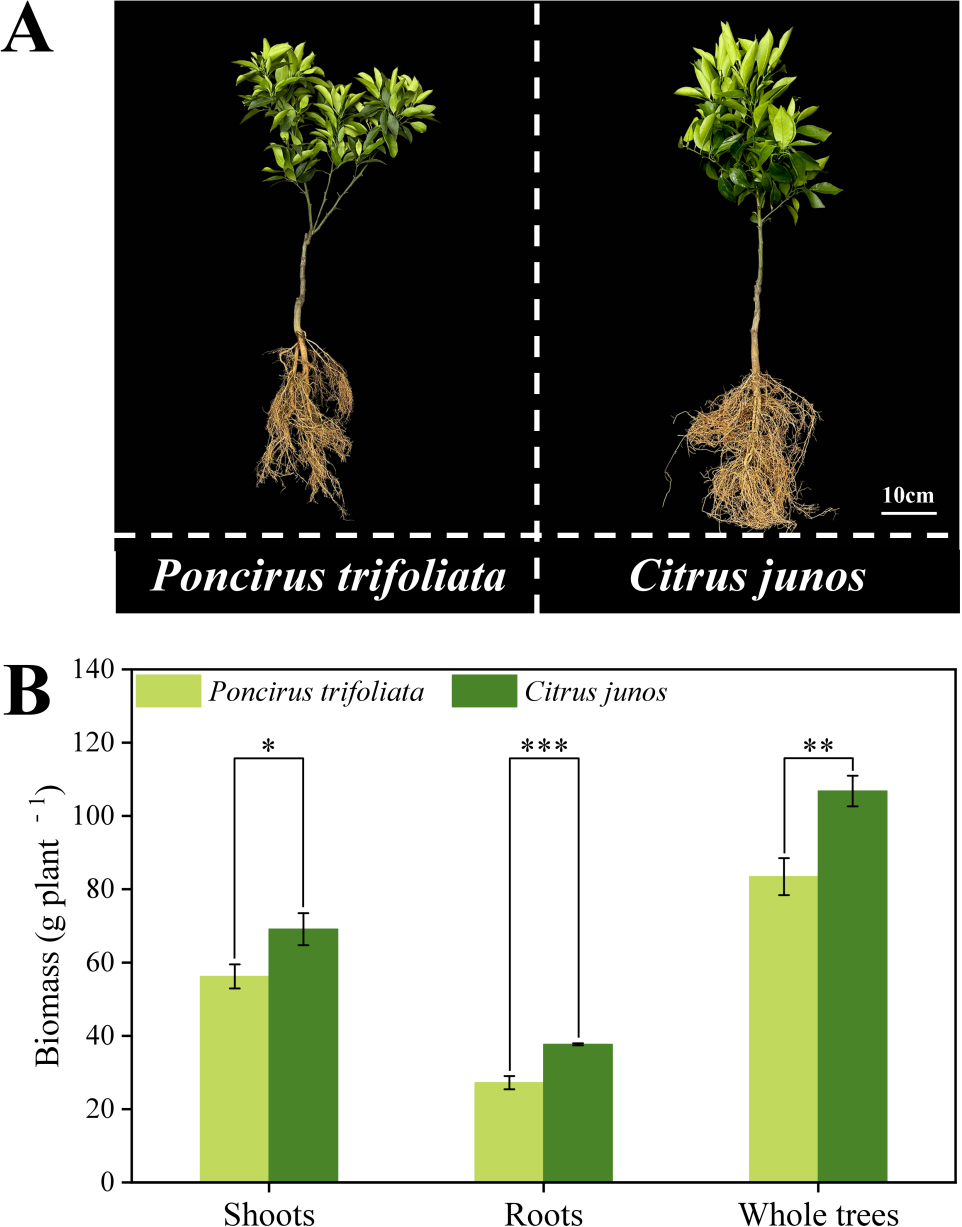
Effect of different rootstocks on growth of top grafted citrus trees. **(A)** represents the phenotype of top grafted citrus trees with different rootstocks. **(B)** represents the biomass (g plant^-1^) of the citrus trees. Single asterisk (*) indicates significant differences at *p* < 0.05, double asterisks (**) indicates significant differences at *p* < 0.01 and triple asterisks (***) indicates significant differences at *p* < 0.001.

The growth of citrus trees top-grafted on *C. junos* (Sieb.) Tanaka rootstock was better than that on *Poncirus trifoliata* (L.) Raf. rootstock (**Figure 2A**). Compared with *Poncirus trifoliata* rootstock, the shoot, root and whole tree biomass of citrus trees top-grafted on *C. junos* were significantly higher by 22.93%, 38.48% and 28.01%, respectively (**Figure 2B**). These results show that citrus top grafted on *C. junos* (Sieb.) Tanaka rootstocks had the better growth performance of all plant parts.

### 3.2 Effects of different rootstocks on Zn nutrition of top grafted citrus

**Figure 3:**
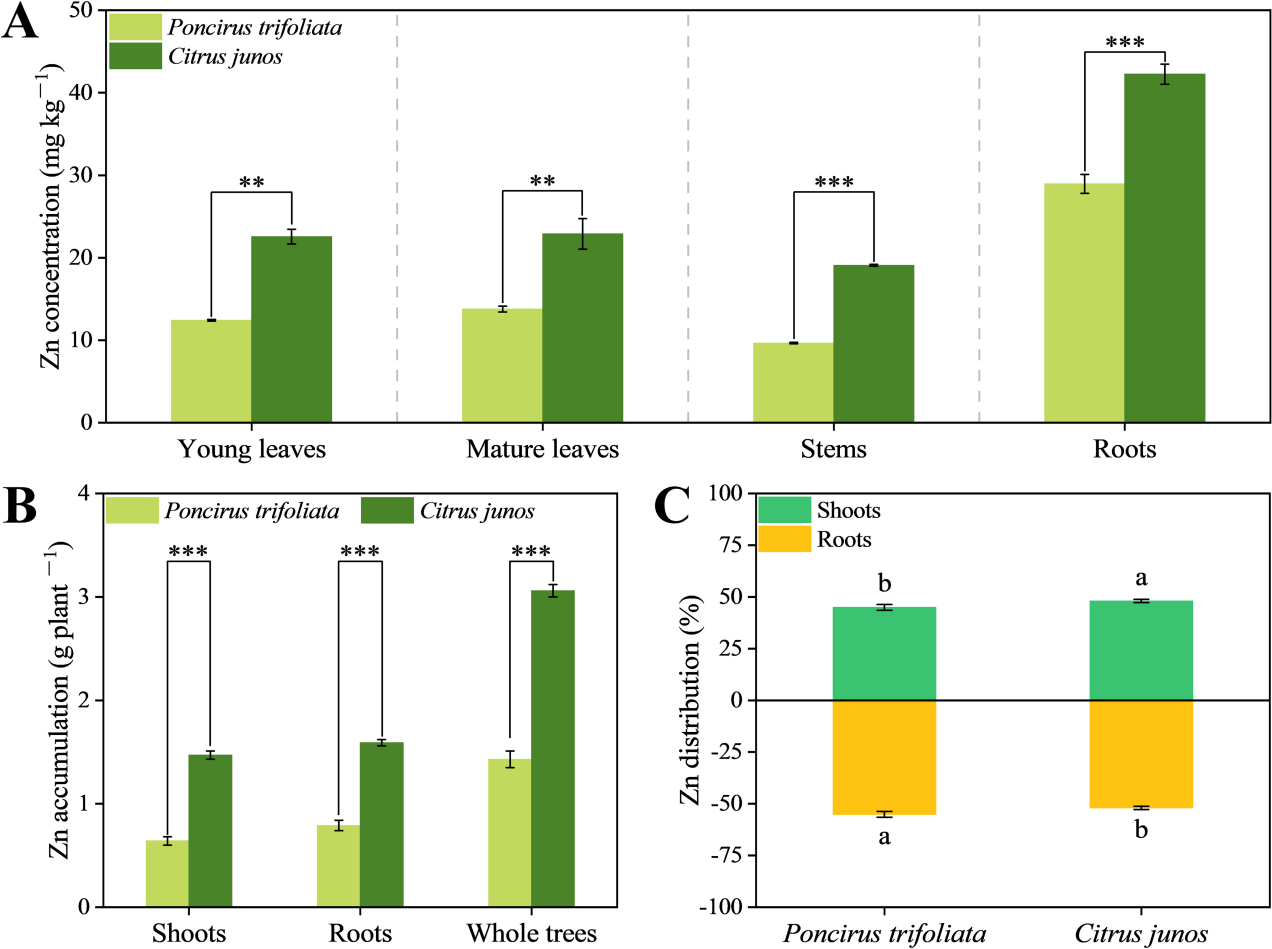
Effect of different rootstocks on zinc nutrition of top grafted citrus trees. **(A)** represents Zn concentrations of young leaves, mature leaves, stems, and roots. **(B)** represents Zn accumulation in shoots, roots, and whole trees. **(C)** represents the Zn distribution between shoots and roots. Single asterisk (*) indicates significant differences at *p* < 0.05, double asterisks (**) indicates significant differences at *p* < 0.01 and triple asterisks (***) indicates significant differences at *p* < 0.001. Different lowercase letters represent statistically significant differences between different rootstocks.

Compared with *Poncirus trifoliata* (L.) Raf. rootstocks, *C. junos* (Sieb.) Tanaka rootstocks significantly increased the Zn concentrations of young leaves, mature leaves, stems, and roots by 81.69%, 66.18%, 97.52%, and 45.94%, respectively (**Figure 3A**). In addition, the aboveground, root and whole tree Zn accumulation of citrus trees top-grafted on *C. junos* rootstocks were significantly higher than for citrus trees top-grafted on *Poncirus trifoliata* rootstock by 128.99%, 102.13% and 114.21%, respectively (**Figure 3B**). In citrus trees top grafted on *C. junos* rootstocks, total Zn partitioning was increased in favor of aboveground tissues by 6.89% compared to *Poncirus trifoliata* rootstocks (**Figure 3C**). Thus, *C. junos* (Sieb.) Tanaka rootstocks were more favorable for the absorption and utilization of Zn.

### 3.3 Effects of different rootstocks on gas exchange parameters of top grafted citrus leaves

**Figure 4:**
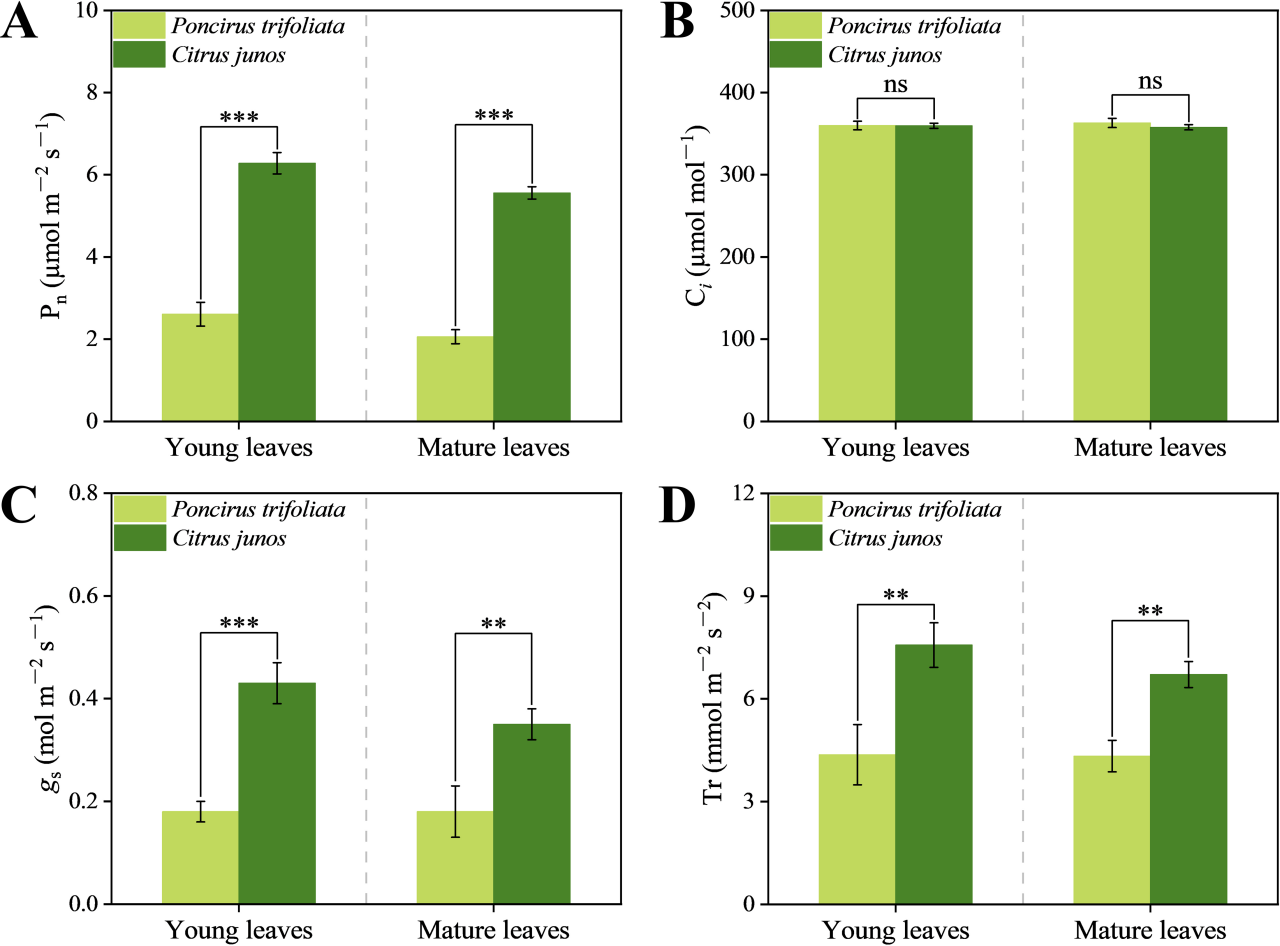
Effect of different rootstocks on gas exchange parameters in young and mature leaves of top grafted citrus trees. **(A–D)** represent the net photosynthetic rate (Pn), intercellular CO_2_ concentration (C*_i_*), stomatal conductance (*g*_s_), and transpiration rate (Tr). Double asterisks (**) indicates significant differences at *p* < 0.01 and triple asterisks (***) indicates significant differences at *p* < 0.001, ns indicates no significant difference.

Compared with *Poncirus trifoliata* (L.) Raf. rootstocks, *C. junos* (Sieb.) Tanaka rootstocks significantly increased net photosynthetic rate (P_n_), stomatal conductance (*g*_s_), and transpiration rate (Tr) of young and mature leaves by 140.18%, 136.39%, and 73.23%, and by 169.74%, 99.94% and 55.04%, respectively (**Figures 4A, C, D**). In general, *C. junos* (Sieb.) Tanaka rootstock has a positive effect on photosynthetic efficiency of top-grafted citrus.

### 3.4 Effects of different rootstocks on photosynthetic pigment concentration of top grafted citrus leaves

**Figure 5:**
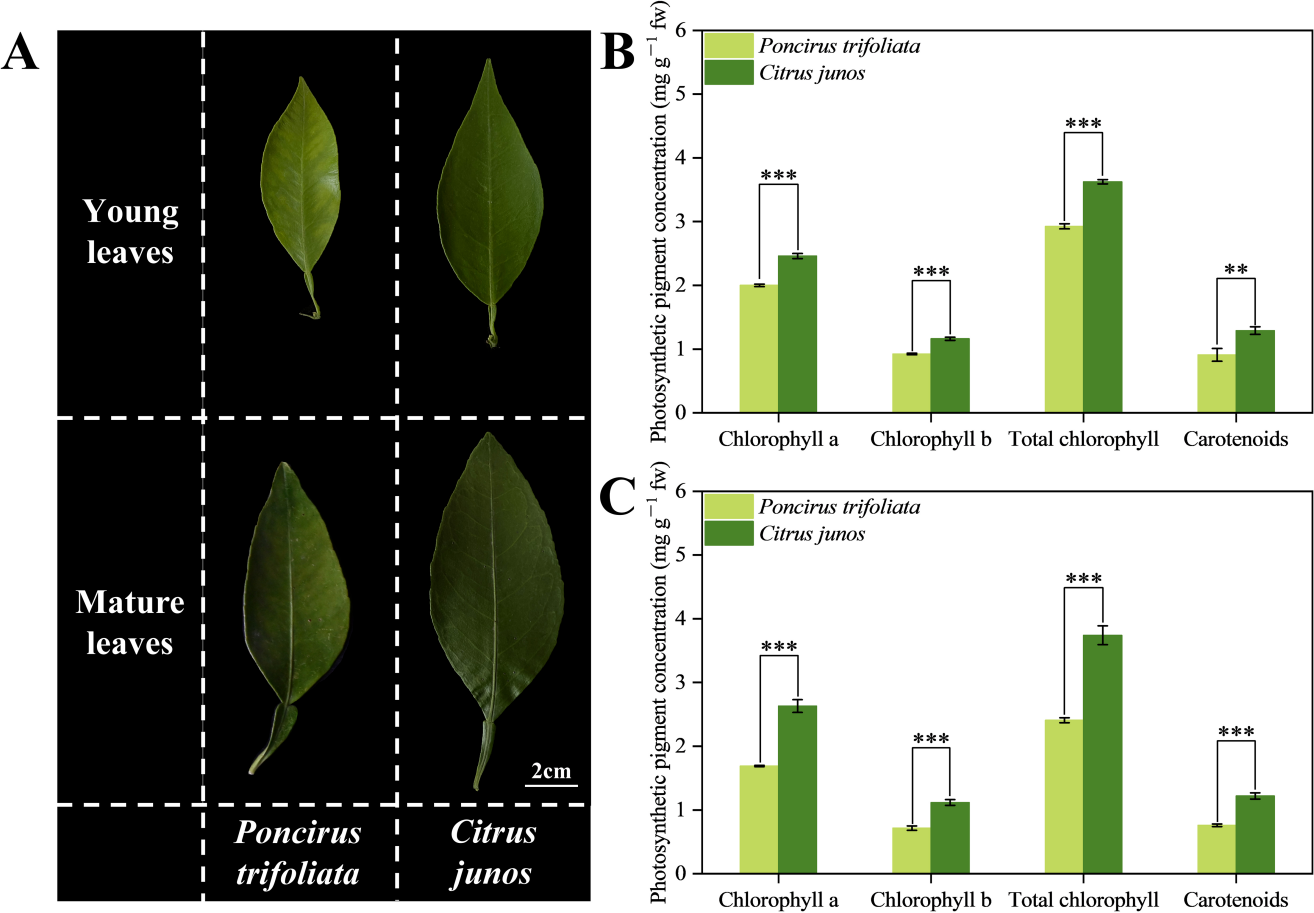
Effect of different rootstocks on photosynthetic pigment concentrations in young and mature leaves of top grafted citrus trees. **(A)** represent leaves phenotypes. **(B)** represents young leaves photosynthetic pigment concentrations. **(C)** represents the mature leaves photosynthetic pigment concentrations. Double asterisks (**) indicates significant differences at *p* < 0.01 and triple asterisks (***) indicates significant differences at *p* < 0.001.

Young leaves of citrus trees top-grafted on *Poncirus trifoliata* rootstocks exhibited severe zinc deficiency mediated chlorisis (**Figure 5A**), whereas mature leaves showed only mild zinc deficiency symptoms. Compared with *Poncirus trifoliata* (L.) Raf. rootstocks, *C. junos* (Sieb.) Tanaka rootstocks significantly increased chlorophyll a, chlorophyll b, total chlorophyll, and carotenoids concentrations of young leaves by 22.96%, 25.82%, 23.93%, and 42.64%, respectively (**Figure 5B**). *C. junos* rootstocks significantly increased chlorophyll a, chlorophyll b, total chlorophyll, and carotenoids concentrations of mature leaf by 55%, 56.16%, 55.38%, and 60.09%, respectively (**Figure 5C**).

### 3.5 Effects of different rootstocks on carbon and nitrogen partitioning of young and mature leaves on top grafted citrus trees

**Figure 6:**
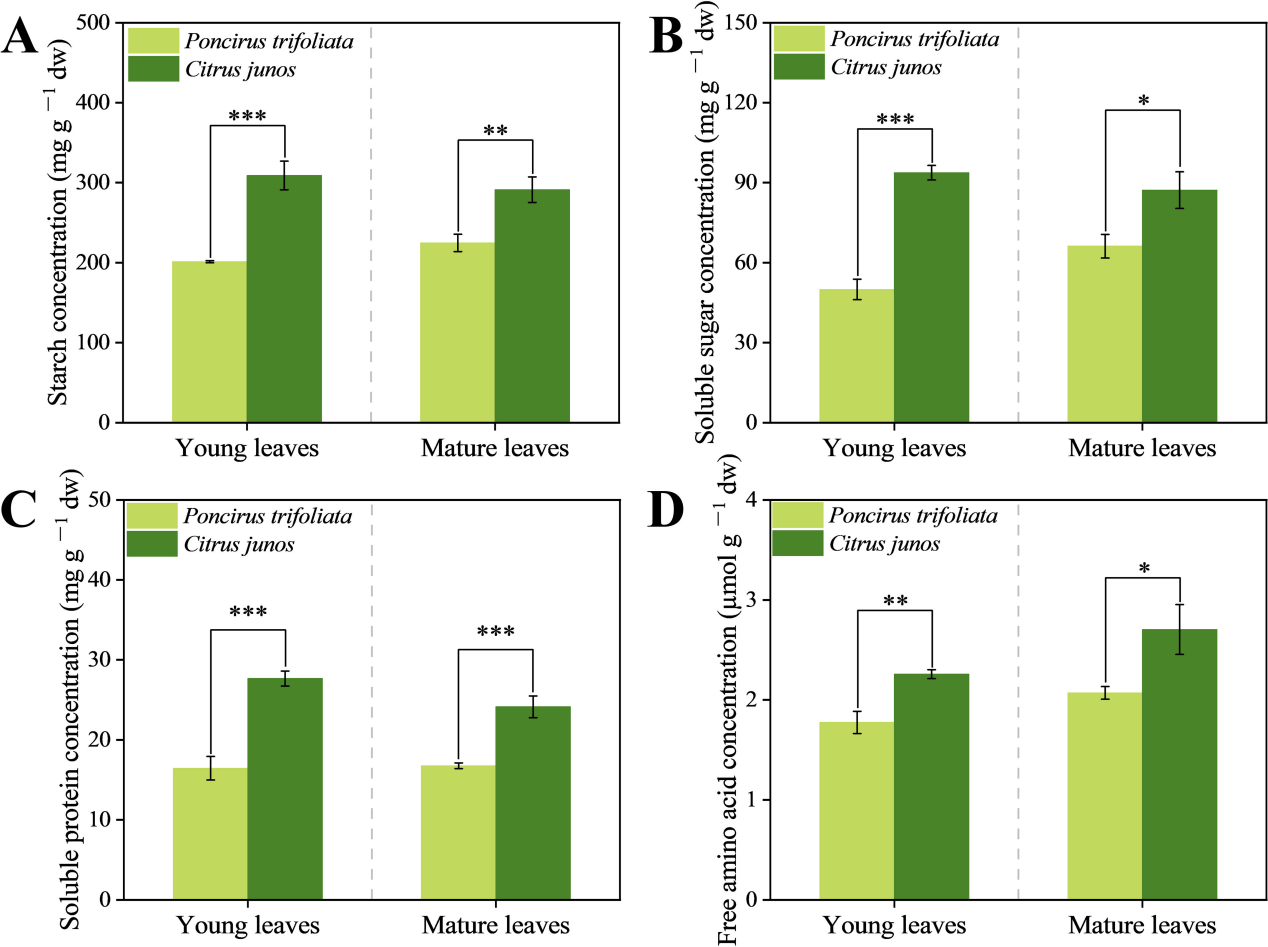
Effect of different rootstocks on carbon and nitrogen partitioning in young and mature leaves of top grafted citrus trees. **(A–D)** represent starch, soluble sugar, soluble protein and free amino acids concentration in leaves respectively. Single asterisk (*) indicates significant differences at *p* < 0.05, double asterisks (**) indicates significant differences at *p* < 0.01 and triple asterisks (***) indicates significant differences at *p* < 0.001.

Compared with *Poncirus trifoliata* (L.) Raf. rootstocks, the *C. junos* (Sieb.) Tanaka rootstocks significantly increased starch, soluble sugar, soluble protein and free amino acid concentrations of young and mature leaves by 53.53%, 87.55%, 68.08% and 27.16%, and by 29.63%, 31.71%, 43.93%, and 30.65%, respectively (**Figure 6**). Thus, *C. junos* (Sieb.) Tanaka Rootstocks better supported C and N nutrition of the citrus trees.

### 3.6 Non-targeted metabolome profile analysis of young and mature citrus leaves on top grafted citrus trees

**Figure 7:**
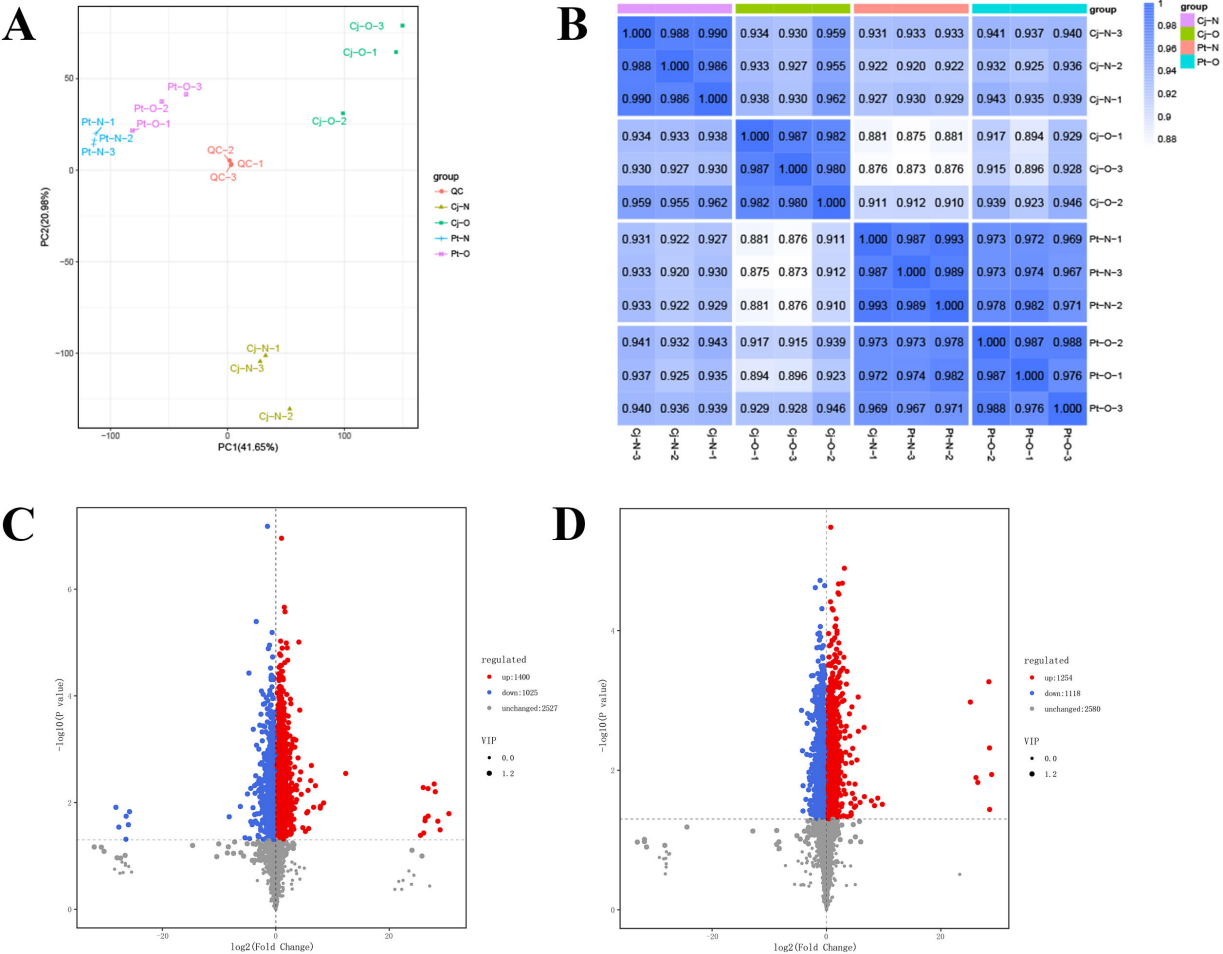
Non-targeted metabolome analyses of citrus leaf metabolites. **(A)** represents principal component analysis (PCA) of all samples. **(B)** represents the correlation between young and mature citrus leaves at different rootstock. **(C, D)** represent volcano plots of differential metabolites in young leaves and mature leaves, respectively. Pt-N: young leaves of citrus trees top-grafted with *Poncirus trifoliata* (L.) Raf. rootstock; Pt-O: mature leaves of citrus trees top-grafted with *Poncirus trifoliata* (L.) Raf. rootstock; Cj-N: young leaves of citrus trees top-grafted with *C. junos* (Sieb.) Tanaka rootstock; Cj-O: mature leaves of citrus trees top-grafted with *C. junos* (Sieb.) Tanaka rootstock.

To identify key metabolites and pathways involved in carbon and nitrogen partitioning of young and mature leaves on citrus trees, top grafted on different rootstocks, we conducted non-targeted metabolome analysis (**Figure 7**). The score of the first principal component (PC1) was 41.65%, the score of the second principal component (PC2) 20.98% (**Figure 7A**). This results indicates separation of metabolite concentrations between the different rootstocks. The heat-map showed that the replicate samples for different rootstocks are tightly clustered, indicating good data reproducibility. By analyzing the correlations between samples, we found good replicability within the groups (**Figure 7B**).

We identified a total of 4952 differentially accumulated metabolites. A total of 2425 differentially accumulated metabolites were identified in the comparison between young leaves on plants top-grafted with *C. junos* (Sieb.) Tanaka rootstocks and *Poncirus trifoliata* (L.) Raf. Rootstocks, with 1400 being upregulated and 1025 being downregulated (**Figure 7C**). In the comparison of mature leaves, a total of 2372 differentially accumulated metabolites were identified between plants with different rootstocks, with 1254 up-regulated and 1118 down-regulated (**Figure 7D**).

### 3.7 Differentially accumulated metabolite enrichment analysis

**Figure 8:**
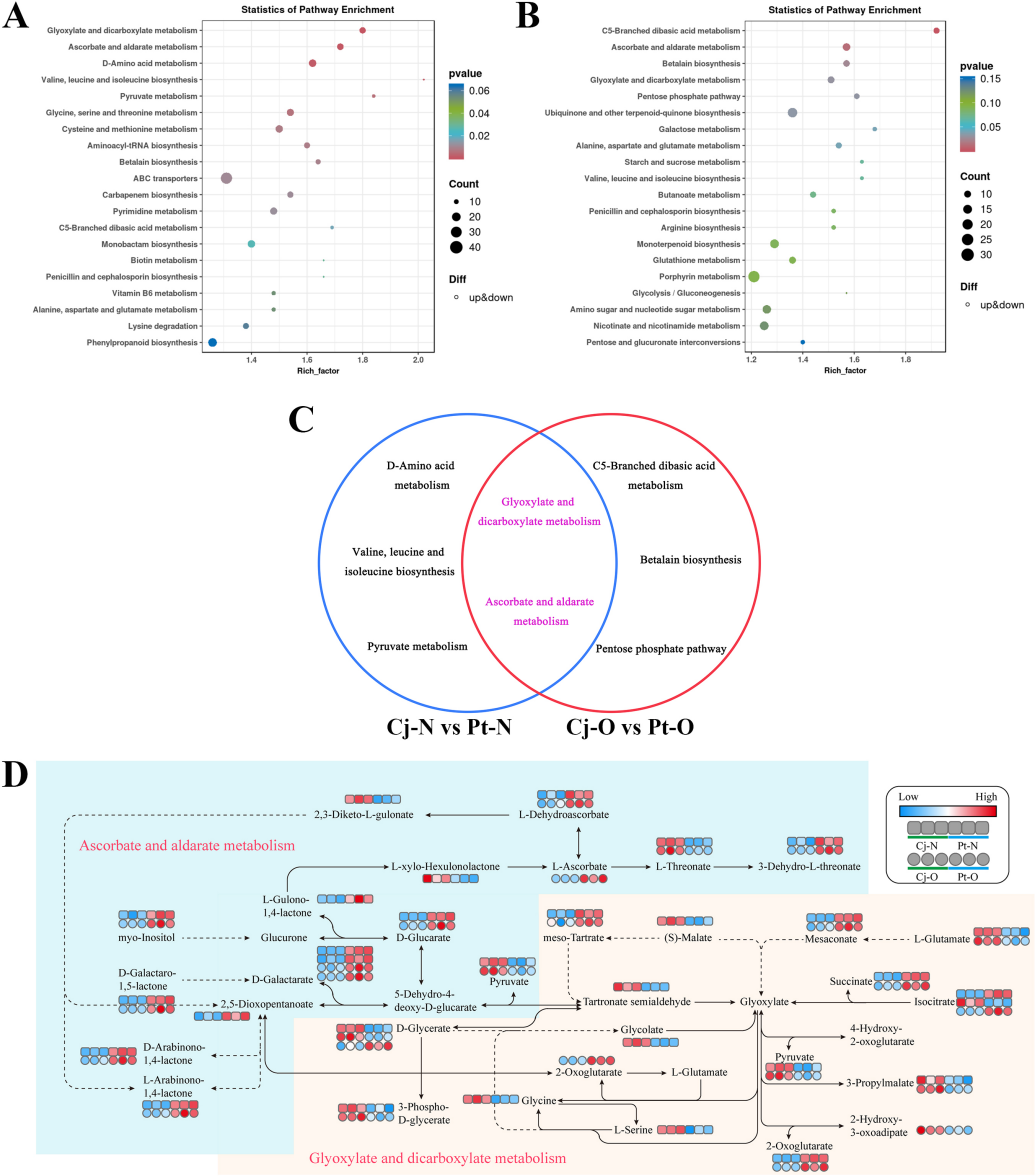
Metabolic pathway profile analyses of young and mature leaves of citrus shoots top grafted on different rootstocks. **(A, B)** represent the KEGG enrichment analyses of differential metabolites in young and mature citrus leaves, respectively. **(C)** represents the Venn diagram of top 5 differential metabolic pathways between young and mature citrus leaves. **(D)** represents the glyoxylate and dicarboxylate metabolism pathway and the ascorbate and aldarate metabolism pathway. In the metabolic pathway maps, red dots represents up-regulated metabolites, blue dots represents down-regulated metabolites. Pt-N: young leaves of citrus trees top-grafted with *Poncirus trifoliata* (L.) Raf. rootstock; Pt-O: mature leaves of citrus trees top-grafted with *Poncirus trifoliata* (L.) Raf. rootstock; Cj-N: young leaves of citrus trees top-grafted with *C. junos* (Sieb.) Tanaka rootstock; Cj-O: mature leaves of citrus trees top-grafted with *C. junos* (Sieb.) Tanaka rootstock.

The differentially accumulated metabolites selected from each comparison group were matched to the KEGG database to obtain the pathways involved in these enrichments. We selected the top 20 pathways with *P*-values in ascending order to draw bubble charts. In the comparison group of young leaves (**Figure 8A**), 16 differentially accumulated metabolites were enriched in gyoxylate and dicarboxylate metabolism (ko00630), 17 in ascorbate and aldarate metabolism (ko00053), 20 in D-Amino acid metabolism (ko00470), 9 in valine, leucine and isoleucine biosynthesis (ko00290), and 10 in pyruvate metabolism (ko00620). In the comparison group of mature leaves (**Figure 8B**), 11 differentially accumulated metabolites were enriched in C5-Branched dibasic acid metabolism (ko00660), 15 in ascorbate and aldarate metabolism (ko00053), 12 in betalain biosynthesis (ko00965), 13 in glyoxylate and dicarboxylate metabolism (ko00630), and 10 in the pentose phosphate pathway (ko00030). Analysis of the top 5 pathways of significant enrichment in the comparison groups of young and mature leaves revealed that differentially accumulated metabolites in both comparisons were enriched in glyoxylate and dicarboxylate metabolism (ko00630) and ascorbate and aldarate metabolism (ko00053) (**Figure 8C**).

Compared to plants with *Poncirus trifoliata* (L.) Raf. rootstocks, most of the differentially accumulated metabolites in the leaves of citrus plants top-grafted on *C. junos* (Sieb.) Tanaka rootstocks were significantly up-regulated in the glyoxylate and dicarboxylate metabolism (ko00630) pathways (**Figure 8D**). Among these metabolites, pyruvate, D-glycerate, 3-phospho-D-glycerate, 3-propylmalate and L-glutamate were significantly up-regulated in both young and mature leaves. In addition, (S)-malate, glycolate, glycine and L-serine were significantly up-regulated in young leaves, while 2-hydroxy-3-oxopropanoate was significantly up-regulated in mature leaves. Compared with citrus plants on *Poncirus trifoliata* (L.) Raf. rootstocks, most of the down-regulated differentially accumulated metabolites in the leaves of citrus plants top-grafted on *C. junos* (Sieb.) Tanaka rootstocks were enriched in the ascorbate and aldarate metabolism (ko00053) pathways (**Figure 8D**). Among these metabolites L-dehydrosorbate, 3-dehydro-L-threonate, myo-inositol, D-glucarate, D-galactarate, D-galactaro-1,5-lactone, D-arabinono-1,4-lactone and L-arabinono-1,4-lactone were significantly down-regulated in both young and mature leaves In addition, L-gulono-1,4-lactone and 2,5-dioxopentanoate were significantly down-regulated in young leaves, while L-ascorbate was significantly down-regulated in mature leaves.

### 3.8 Correlation of Zn concentration, physiological parameters, and metabolites in leaves

As shown in **Supplementary Figure S1**, Zn concentration positively correlated with the physiological parameters analyzed in citrus leaves except for intercellular CO_2_ concentration (C*_i_*). In both, young and mature leaves, Zn concentrations were positively correlated with pyruvate, D-glycerate, 3-phospho-D-glycerate, 3-propyImalate and L-glutamate, while negatively correlated with L-dehydrosorbate, 3-dehydro-L-threonate, myo-inositol, galactaric acid, D-galactaric acid, D-galactaro-1,5-lactone, D-arabinono-1,4-lactone and L-arabinono-1,4-lactone. In addition, in young leaves, (S)-malate (*P*<0.01), glycolate (*P*<0.001), glycine (*P*<0.001), and L-serine (*P*<0.01) were significantly positively correlated with Zn concentrations, while D-glucarate (*P*<0.001), L-gulono-1,4-lactone (*P*<0.001), and 2,5-dioxopentanoate (*P*<0.01) were significantly negatively correlated with Zn concentrations (**Supplementary Figure S1A**). In mature leaves, 2-hydroxy-3-oxopropanoate (*P*<0.001) was significantly positively correlated with the Zn concentration (**Supplementary Figure S1B**).

## 4 Discussion

This study highlights the critical impact of rootstock selection on the growth, nutrient uptake, and metabolic processes of top-grafted citrus trees. *Citrus junos* rootstock significantly improves plant growth, Zn nutrition, photosynthesis, and foliar carbon and nitrogen accumulation, while alleviating Zn deficiency symptoms observed in leaves of top grafted citrus. Moreover, metabolomic analysis reveals that Zn deficiency in *P. trifoliata* rootstock impairs key metabolic pathways, including carbon and nitrogen metabolism, while triggering antioxidant defenses. These findings underscore the importance of selecting appropriate rootstocks, such as *C. junos*, to enhance nutrient efficiency, metabolic balance, and plant resilience under challenging soil conditions.

### 4.1 Effects of rootstocks on the growth of top-grafted citrus trees

Consistent with our hypothesis (i), different rootstocks lead to significant differences in growth and leaf phenotypes of citrus plants. Citrus plants grafted on *Poncirus trifoliata* rootstock exhibit poor growth (**Figure 2A**), and display Zn deficiency symptoms such as chlorosis in the leaves (**Figure 4A**). This indicates that selecting appropriate rootstocks can alleviate weakened growth caused by top grafting. Also previous studies showed that different rootstocks have a significant impact on the growth and development of grafted plants (Zhu et al., 2020; Castle, 2010). Citrus plants grafted on *Poncirus trifoliata* rootstocks exhibited inhibited growth in zinc-deficient soils (Chen et al., 2014) and nutrient deficiency and stunted growth in alkaline soils (Wu et al., 2019). *Poncirus trifoliata* (L.) Raf. has short or only few root hairs under field conditions, which limits its nutrient absorption (Wu et al., 2011). *C. junos* rootstocks can regulate root hormone signaling pathways to help the roots adapt to alkaline environments, thus maintaining normal plant growth (Wu et al., 2019). As a consequence, Citrus plants grafted on *C. junos* rootstocks exhibit significantly higher shoot biomass, root biomass, and total biomass compared to those on *Poncirus trifoliata* rootstocks (**Figure 2B**).

### 4.2 Effects of rootstocks on Zn nutrition of top-grafted citrus trees

Rootstock types have been reported to affect foliar mineral element concentrations of grafted trees (Mattos et al., 2003; Toplu et al., 2008). This may be related to differences in the root architecture, such as deep and medium branching, and their capacity for nutrient uptake (Ghimire et al., 2023). In the present study, different rootstocks induced variations in Zn nutrition in top-grafted citrus trees (**Figure 3**), consistent with our hypothesis (i),. This indicates that replacing *Poncirus trifoliata* rootstocks with *C. junos* rootstocks can counteract Zn deficiency in citrus plants top-grafted under alkaline conditions. Differences in Zn concentrations of citrus leaves induced by rootstocks have already been reported (Yilmaz et al., 2018). In alkaline soils, when *Poncirus trifoliata* is used as rootstock, Zn absorption is very low, whereas *C. junos* rootstocks can absorb Zn at higher levels (Balal et al., 2011; Rodríguez-Gamir et al., 2010). This difference in Zn acquisition can be ascribed to the upregulation of Zn transport-related genes, such as ZIP1, ZIP5, and ZIP10, in *C. junos* under alkaline conditions (Wu et al., 2019). The present study also show that *C. junos* rootstocks significantly enhance Zn allocation to the shoots (**Figures 3B, C**). Previous study found that a Zn/Fe-regulated transporter (ZRT/IRT)-related protein (ZIP) gene was significantly induced in leaves and roots of Zn-deficient trifoliate orange plants (Fu et al., 2017). Therefore, rootstock-specific regulation of Zn transporters, such as ZIP genes, may contribute to the enhanced Zn uptake and transport observed in *C. junos*, though this remains to be experimentally confirmed. Notably, Zn plays a crucial role in enhancing fruit yield and quality (Arshad et al., 2024). This study also provides a theoretical basis for improving fruit quality.

### 4.3 Effects of rootstocks on leaf physiology of top-grafted citrus trees

Consistent with our hypothesis (ii), different rootstocks induced variations in photosynthetic efficiency and carbon-nitrogen accumulation in grafted citrus leaves (**Figures 4**-**6**). *C. junos* rootstocks enhanced the net photosynthetic rate (**Figure 4A**), stomatal conductance (**Figure 4C**), and transpiration rate (**Figure 4D**) of the leves. Apparently, the rootstock affects the photosynthetic efficiency, energy metabolism, and protein synthesis of leaves of top-grafted citrus trees. Previous studies indicated that zinc enhances K^+^ influx into guard cells, leading to improved stomatal conductance and transpiration efficiency (Hassan et al., 2020). Moreover, Zn acts as a cofactor for photosynthetic enzymes like carbonic anhydrase and RuBisCO, thereby enhancing CO_2_ fixation efficiency and increasing net photosynthetic rate (Dang et al., 2024).

Compared to *Poncirus trifoliata* rootstock, *C. junos* rootstock significantly increased the photosynthetic pigment concentration in the leaves of top-grafted citrus trees (**Figures 5B, C**). Also in previous studies, the chlorophyll concentration of leaves in citrus on *C. junos* rootstocks was higher than on *Poncirus trifoliata* rootstock (Yin et al., 2021; Zhu et al., 2021). This finding may be related to the differences in Zn deficiency-induced chlorosis, as Zn is an essential cofactor for enzymes involved in chlorophyll synthesis (Hussain et al., 2021). The *C. junos* (Sieb.) Tanaka rootstock increased the sugar levels in top-grafted citrus leaves (**Figures 6A, B**). In previous studies of *Tamarix chinensis* and tobacco, this effect was attributed to increased chlorophyll concentrations (Sun et al., 2021; Chen et al., 2016) that improved photosynthesis and positively affecting sugar production in leaves (Pacholczak and Nowakowska, 2020). Carbohydrates not only serve as energy reserves but also provide carbon skeletons for nitrogen assimilation into amino acids, and further on into proteins (Baslam et al., 2021). Consequently, our study found higher levels of free amino acids and protein in the leaves of top-graft citrus trees using *C. junos* (Sieb.) Tanaka as the rootstock (**Figures 6C, D**). Although fruit quality was not evaluated in this study, previous research has shown that *C. junos* rootstock can promote amino acid biosynthesis and increase organic acid content in citrus fruits (Xiong et al., 2023; Wang et al., 2024), both of which are key contributors to improved fruit flavor and nutritional quality. Based on this characteristic of the *C. junos* rootstock, our research group has also been continuously conducting studies on fruit quality and amino acid metabolism in citrus trees grafted onto *C. junos* (Zhao et al., 2025).

### 4.4 Effects of rootstocks on leaf metabolite enrichment analysis

In this study, metabolomics analysis revealed that most of the metabolites in the glyoxylate and dicarboxylate metabolism pathway (ko00630) in citrus leaves top-grafted on *Poncirus trifoliata* rootstock were significantly downregulated (**Figure 8D**), with both young and mature leaf showing signs of Zn deficiency. Due to their higher metabolic activity, young leaves require more Zn, leading to a greater reduction in metabolites, whereas mature leaves, having lower metabolic demands, are less affected by Zn deficiency. Zn acts as an essential cofactor for many enzymes in carbon and nitrogen metabolism, participating in key reactions in glycolysis, the tricarboxylic acid cycle (TCA cycle), and the glyoxylate pathway (Yang et al., 2023). Additionally, Zn is crucial for amino acid synthesis (Barrameda-Medina et al., 2017). Therefore, metabolomic analysis revealed low abundances of glycine and L-serine in zinc-deficient citrus leaves induced by *Poncirus trifoliata* rootstock. Glycine is a critical intermediate in the photorespiratory pathway (Jiang et al., 2023), and its reduced levels can restrict photorespiration, thereby suppressing photosynthesis and limiting carbohydrate synthesis. Moreover, studies have shown that zinc deficiency reduces the activity of enzymes such as pyruvate kinase and malate dehydrogenase in plants (Wang et al., 2019; Navarro-León et al., 2016), leading to decreased accumulation of metabolites including pyruvate, malate, and 3-propylmalate. In summary, Zn deficiency limited carbon dioxide fixation and decreased the accumulation of non-structural carbohydrates in leaves. It also reduced the production of 3-phospho-D-glycerate, which in turn suppressed glycolysis and led to decreased pyruvate levels. This also limited the entry of pyruvate into the tricarboxylic acid (TCA) cycle, thereby reducing the production of intermediates such as malate and oxaloacetate. These metabolites are not only key nodes in energy metabolism, but also provide carbon skeletons for amino acid synthesis (Morley et al., 2023; Zhu et al., 2021). Their reduction further inhibited amino acid and protein biosynthesis, which supported our physiological findings.

Metabolome analysis also revealed that numerous metabolites in the ascorbate and aldarate metabolism pathway were significantly upregulated in citrus leaves top-grafted onto *Poncirus trifoliata* rootstock, with L-dehydroascorbate, 3-dehydro-L-threonate, myo-Inositol, D-glucarate, and D-galactarate notably increased in both young and mature leaves (**Figure 8D**). These metabolites play crucial roles in antioxidant pathways, essential to mitigate oxidative stress (Xu et al., 2018; Gu et al., 2024). Meanwhile, since Zn is a cofactor for various antioxidant enzymes, its deficiency results in ROS accumulation and oxidative stress (Ren et al., 2024). In addition, the divergent upregulation of some metabolites reveals the varying metabolic strategies between new and old leaves in their response to Zn deficiency. For instance, the significant upregulation of L-gulono-1,4-lactone and 2,5-dioxopentanoate in young leaves suggests that they respond more rapidly to oxidative stress by enhancing the synthesis of ascorbate precursors or intermediates (Wagschal et al., 2020). In contrast, the increase of L-ascorbate levels in mature leaves may indicate a greater reliance on existing antioxidant substances rather than a rapid response through enhanced synthesis of precursor metabolites. In general, the pronounced increase in L-dehydroascorbate and L-ascorbate indicates that the plant is strengthening its antioxidant defenses to eliminate excessive ROS and sustain cellular redox equilibrium (Wang et al., 2025).

Consistent with our hypothesis (iii), rootstocks significantly effected leaf metabolism in top-grafted citrus trees. Overall, *Poncirus trifoliata* rootstock induced a zinc deficiency response in both young and mature leaves of top-grafted citrus trees, leading to disruptions in carbon and nitrogen metabolism and activation of oxidative stress. In contrast, *C. junos* (Sieb.) Tanaka rootstock maintained better zinc nutrition and metabolic homeostasis.

## 5 Conclusion

The present results demonstrate that *C. junos* (Sieb.) Tanaka rootstocks can enhance not only plant growth and Zn acquisition of top-grafted citrus trees, but also can improve photosynthetic efficiency and carbon-nitrogen accumulation capacity. Leaf metabolome analysis revealed that differences in key compounds of glyoxylate and dicarboxylate metabolism as well as ascorbate and aldarate metabolism pathways responded to the rootstock-induced changes in Zn levels. These results provide scientific support for the selection and optimization of rootstocks for the production of top-grafted citrus trees.

## Data Availability

The raw data supporting the conclusions of this article will be made available by the authors, without undue reservation.
