# Supplementary material for: Nutrient acquisition efficient rootstocks improve zinc nutrition of top-grafted citrus trees on calcareous soil
Source: Front Plant Sci. 2025 Jul 31;16:1615405. doi: 10.3389/fpls.2025.1615405 (PMC12350133; doi:10.3389/fpls.2025.1615405)
Supplement: Supplementary file 1 [file Image1.pdf]

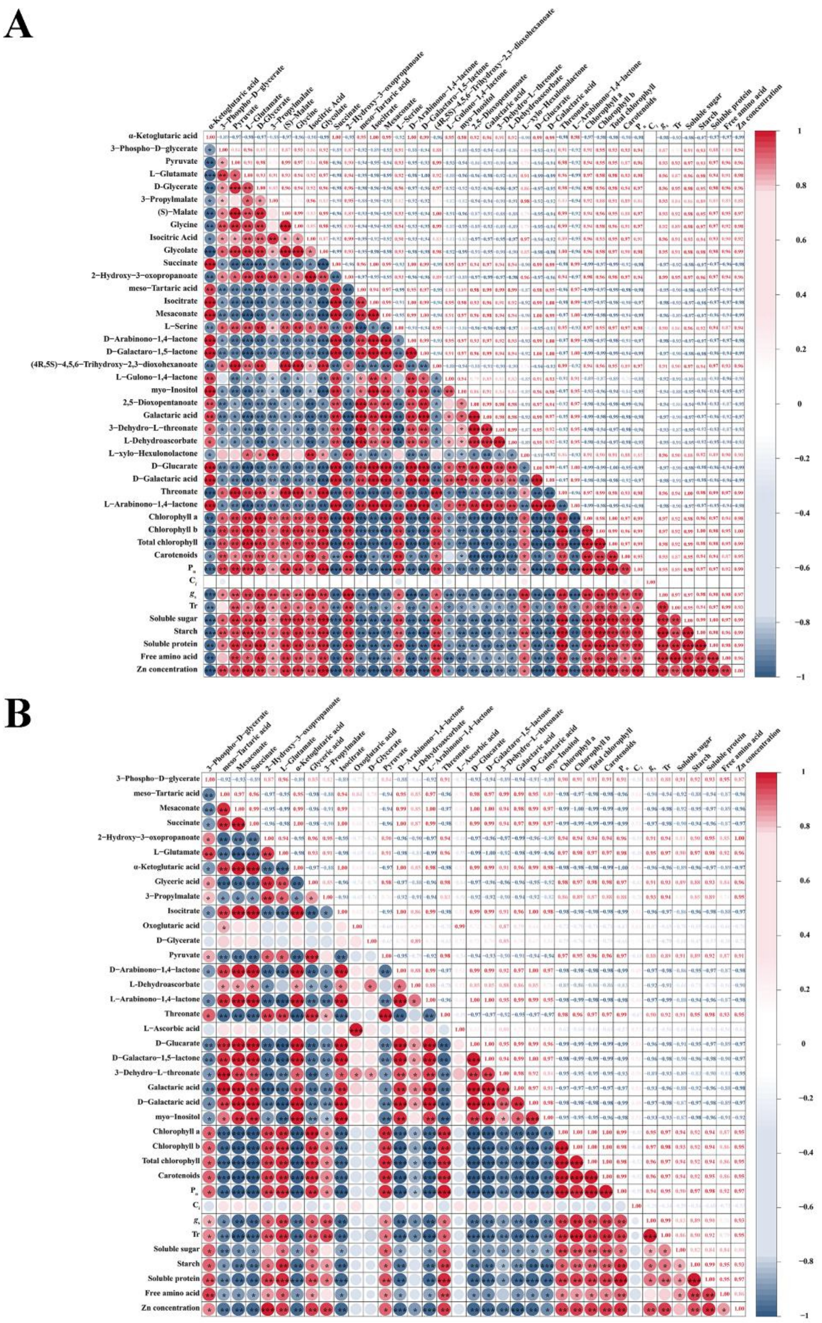

Supplementary Figure 1. Correlation analysis of Zn concentration and metabolites in young (Panel A) and mature (Panel B) leaves of top grafted citrus trees. Pearson's correlation coefficients are presented. Single asterisk (*) indicates significant differences at $p < 0.05$, double asterisks (**) indicates significant differences at $p < 0.01$ and triple asterisks (***) indicates significant differences at $p < 0.001$.
